# Case Report: Repeated esophageal obstruction in a patient with type 3C diabetes mellitus

**DOI:** 10.3389/fendo.2023.1225385

**Published:** 2023-07-28

**Authors:** Katsumasa Koyama, Takatoshi Anno, Haruka Takenouchi, Tomohiko Kimura, Kohei Kaku, Hideaki Kaneto

**Affiliations:** ^1^ Department of General Internal Medicine 1, Kawasaki Medical School, Okayama, Japan; ^2^ Department of Diabetic Medicine, Kurashiki Central Hospital, Kurashiki, Japan; ^3^ Department of Diabetes, Endocrinology and Metabolism, Kawasaki Medical School, Kurashiki, Japan

**Keywords:** esophageal obstruction, type 3C diabetes mellitus, esophageal candidiasis, diabetic neuropathy, gastrointestinal motility disorders

## Abstract

Although diabetic neuropathy is a well-known cause of gastrointestinal motility disorders, it is rare that diabetic neuropathy brings about esophageal obstruction. Here, we report a case with Type 3C diabetes mellitus (DM) lasting over 15 years and repeated esophageal obstruction resulting in chicken-meat-induced esophageal obstruction and candidiasis. This case highlights the importance of management of DM to prevent the development of complications such as diabetic neuropathy and associated symptoms.

## Introduction

Diabetic neuropathy can lead to various impairments in sensory, motor, and autonomic functions, and it is a common complication of diabetes mellitus (DM). The gastrointestinal system is one of the main systems affected by diabetic neuropathy, with up to 75% of patients with DM reporting some kind of long-term gastrointestinal symptoms, including esophageal symptoms (1). The pathogenesis of gastrointestinal dysfunction of diabetic autonomic neuropathy is complex and involves an increase in oxidative stress, changes in gut bacteria, decrease in growth factors, changes in cell signaling pathways, and dysfunction of the endothelium (2).

Poorly glycemic control over a long period of time can lead to autonomic neuropathy, which can cause gastrointestinal motility and sensory disturbances. Type 3C DM can also become a factor for poor glycemic control and may require insulin preparation, similar to type 1 DM, depending on the extent and location of removal of the pancreas (3).

Here, we report a case with Type 3C DM lasting over 15 years and repeated esophageal obstruction resulting in chicken-meat-induced esophageal obstruction and candidiasis. This case highlights the importance of the management of DM to prevent the development of complications such as diabetic neuropathy and associated symptoms.

## Case description

A 72-year-old Japanese male visited the emergency room with symptoms of food obstruction and general fatigue. He had consumed chicken roughly one week prior to the visit and had neglected his symptoms, which worsened over time. Despite having experienced similar symptoms twice in the past at the age of 69 and 71, however, those symptoms improved within a few days. Therefore, he waited to see if his condition would improve for one week. The patient was diagnosed with Type 3C DM at the age of 55, after undergoing a two-thirds pancreatectomy for acute pancreatitis due to excessive drinking (details were unknown). His was treated with 14 units of insulin aspart and 5 units of insulin degludec, along with 0.9 mg/day of voglibose for Type 3C DM management, 20 mg/day of olmesartan and 5 mg/day of amlodipine for hypertension, and 2.5 mg/day of rosuvastatin for dyslipidemia. His height, body weight, and body mass index (BMI) were 152.5 cm, 47.9 kg, and 20.6 kg/m^2^, respectively. As shown in **Table 1**, the patient suffered from severe dehydration, electrolyte imbalance, and renal dysfunction, despite relatively good DM control due to their inability to consume food. He had symptoms of numbness in his legs and the Achilles reflex was absent mainly due to peripheral neuropathy. Additionally, he had symptoms of hypoglycemia unawareness, and orthostatic hypotension mainly due to autonomic neuropathy. He repeatedly suffered from constipation as a lower gastrointestinal motility disorder. The coefficient of variation of R-R intervals (CVR-R) showed autonomic neuropathy as follows: CVR-R(rest), 1.8; CVR-R (breath), 1.9, although we could not examine the nerve conduction studies. Schellong test was positive: blood pressure decreased from 157/98 mmHg (rest) to 126/84 mmHg (3 minutes). He had simple diabetic retinopathy and stage 3 diabetic nephropathy with overt proteinuria.

**Table 1:** Laboratory data observed in the emergence room.

Variable	Result	Reference range	Variable	Result	Reference range
Peripheral blood			Diabetes marker		
White blood cells (/μL)	7200	3300 – 8600	Plasma glucose (mg/dL)	107	
Neutrophil (%)	77.0	52.0 – 80.0	Hemoglobin A1c (%)	7.3	4.9 – 6.0
Eosinophil (%)	0.1	0.0 – 6.0	Total cholesterol (mg/dL)	345	142 – 248
Red blood cells (×10^4^/μL)	511	386 – 492	LDL cholesterol (mg/dL)	188	65 – 139
Hemoglobin (g/dL)	16.7	11.6 – 14.8	HDL cholesterol (mg/dL)	51	40 – 90
Hematocrit (%)	50.5	35.1 – 44.4	Triglyceride (mg/dL)	490	40 – 149
Platelets (×10^4^/μL)	12.5	15.8 – 34.8	Electrolytes		
Blood biochemistry			Sodium (mmol/L)	156	138 – 145
Total protein (g/dL)	8.8	6.6 – 8.1	Potassium (mmol/L)	4.9	3.6 – 4.8
Albumin (g/dL)	5.4	4.1 – 5.1	Chloride (mmol/L)	111	101 – 108
Total bilirubin (mg/dL)	1.0	0.4 – 1.5	IP (mg/dL)	5.5	2.7 – 4.6
AST (U/L)	83	13 – 30	Calcium (mg/dL)	10.7	8.8 – 10.1
ALT (U/L)	48	7 – 23	Magnesium (mg/dL)	3.8	1.9 – 2.6
LDH (U/L)	358	124 – 222	Urinary test		
ALP (U/L)	84	106 – 322	Urinary pH	5.5	5.0 – 7.5
γ-GTP (U/L)	53	9 – 32	Urinary protein	2+	–
BUN (mg/dL)	108.3	8 – 20	Urinary sugar	–	–
Creatinine (mg/dL)	2.15	0.46 – 0.79	Urinary ketone body	–	–
Uric acid (mg/dL)	16.1	2.6 – 5.5	Urinary bilirubin	–	–
Creatine Kinase (U/L)	1197	41 – 153	Urinary blood	1+	–
Amylase (U/L)	711	44 – 132			
CRP (mg/dL)	0.54	<0.14			
BNP (pg/mL)	11.8	0.0 – 18.4			

AST, aspartate aminotransferase; ALT, alanine aminotransferase; LDH, lactate dehydrogenase; ALP, alkaline phosphatase; γ-GTP, γ-glutamyltranspeptidase; BUN, blood urea nitrogen; CRP, C-reactive protein; BNP, brain natriuretic peptide; LDL, Low-density lipoproteins; HDL, High-density lipoprotein; IP, Inorganic Phosphorus.

**Figure 1:**
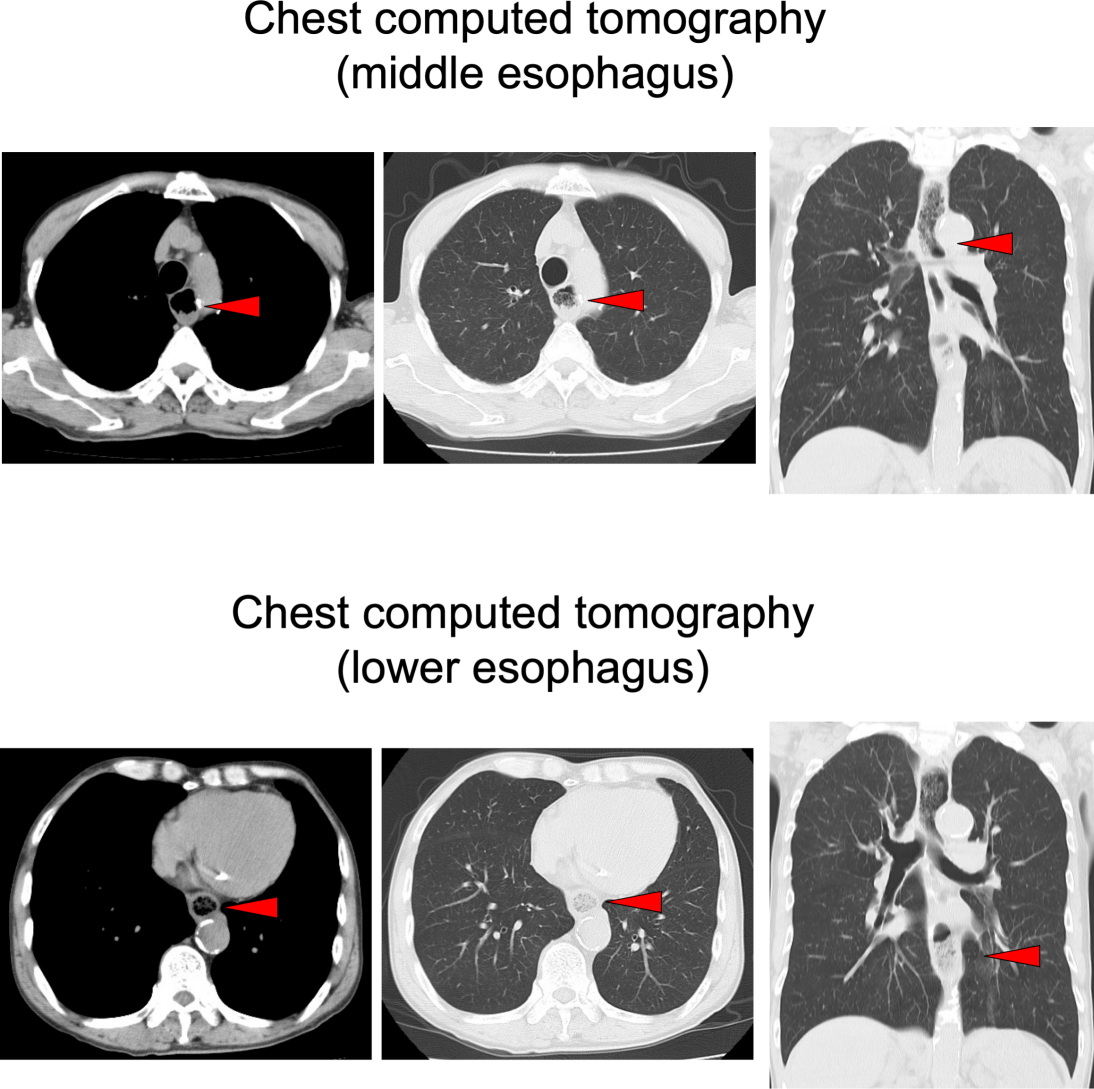
Chest computed tomography in the emergency room. A foreign body (red allow head) was detected in the middle esophagus (upper panels) and it was noted that the space was narrowed with the expansion of the upper lesion of the esophagus. Expansion of the esophagus was also observed in the lower esophagus (lower panels).

**Figure 2:**
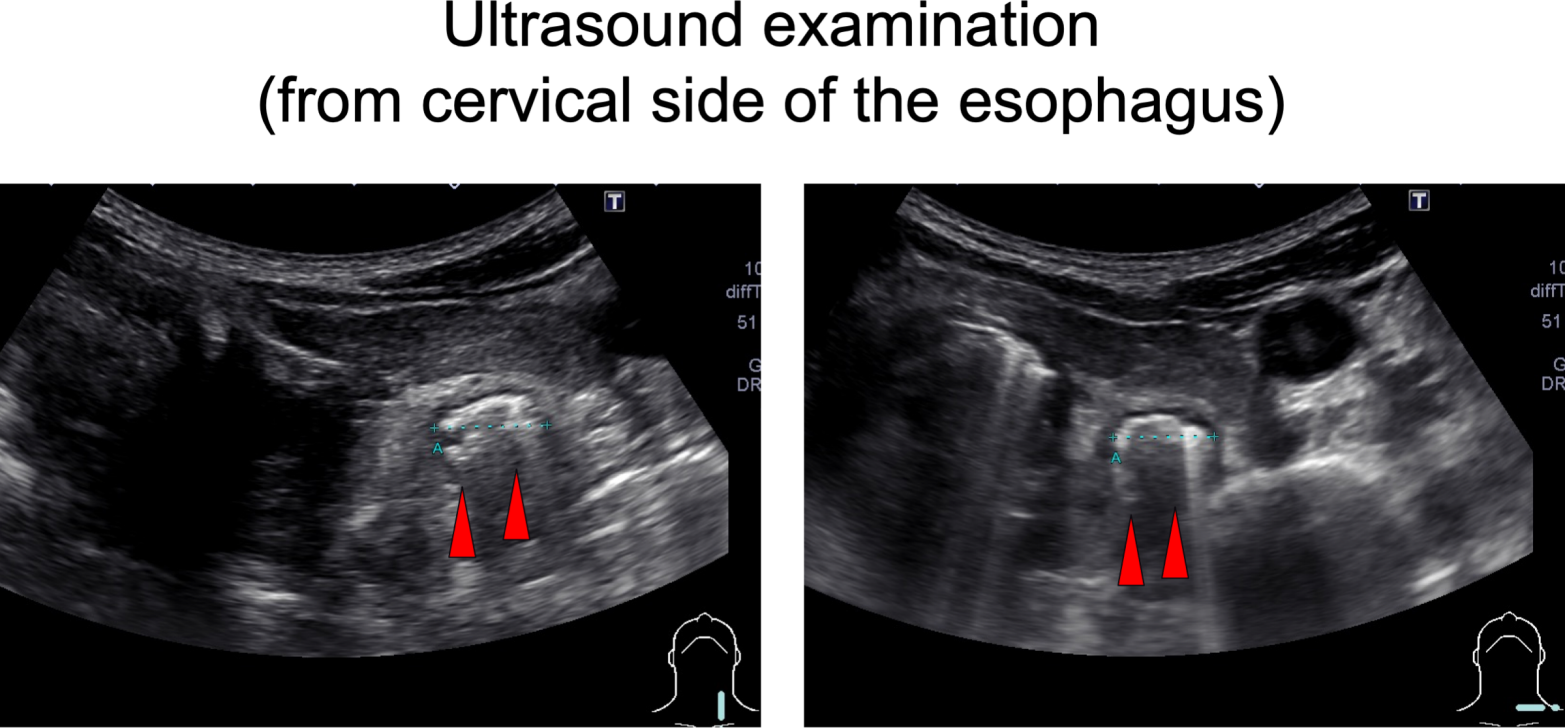
Ultrasound examination. An approximately 1 cm long, high echoic mass was detected in the cervical side of the esophagus (a red arrowhead).

**Figure 3:**
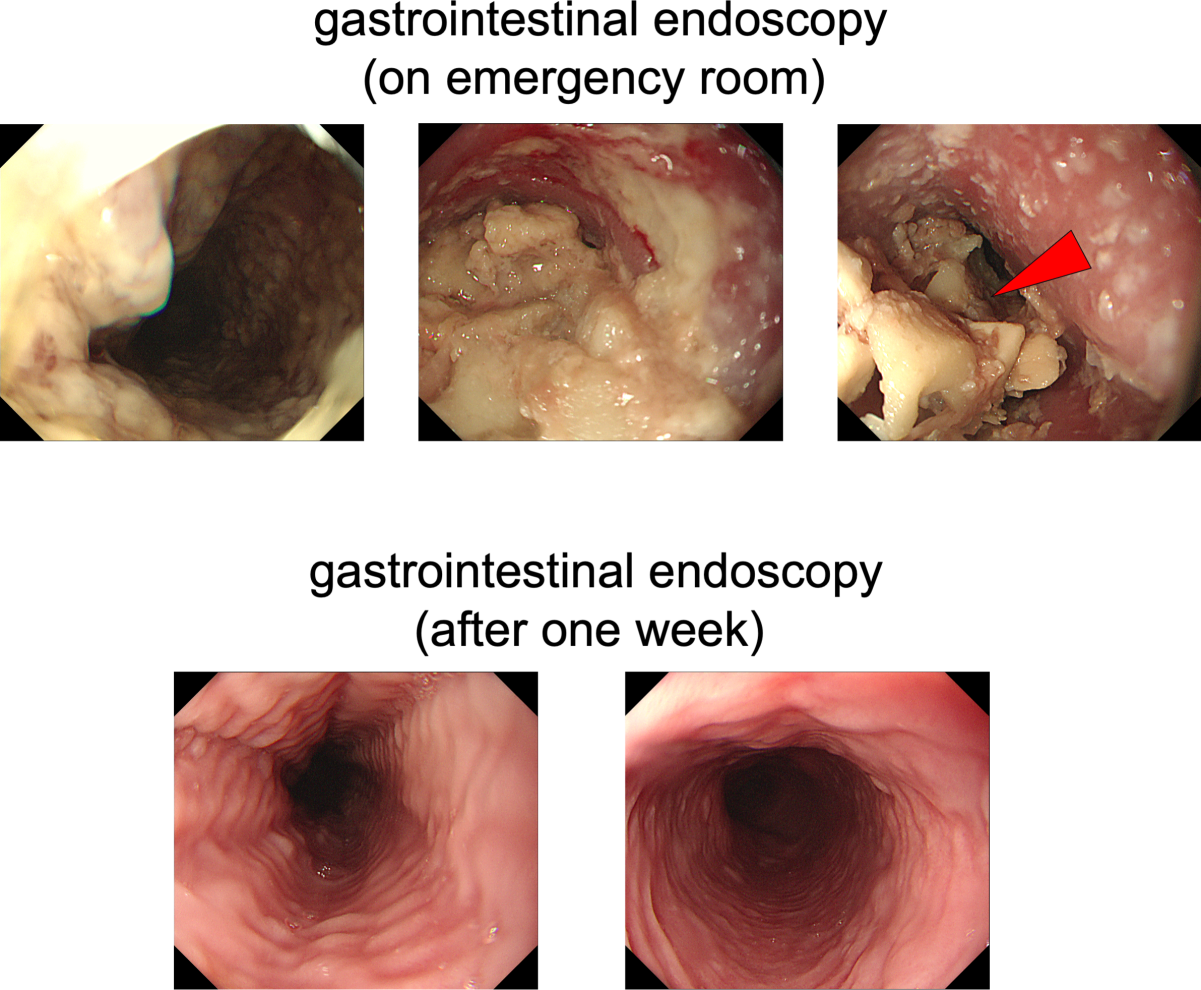
Upper gastrointestinal endoscopy. In the upper panels, circumferential esophageal candidiasis was observed at the entrance and upper part of the esophagus. The middle part of the esophagus was completely obstructed by a piece of chicken meat and other foodstuffs. In the lower panels, a follow-up upper gastrointestinal endoscopy was performed one week later and conducted without any complications.

On chest computed tomography, a foreign body was detected in the middle esophagus (**Figure 1**; upper panels) and it was noted that the space was narrowed with the expansion of the upper lesion of the esophagus. Additionally, expansion was also observed in the lower esophagus (**Figure 1**; lower panels). Ultrasound examination revealed a high-echoic mass in the cervical side of the esophagus, which was suspected to be food residue of approximately 1 cm in length (**Figure 2**). Given the patient’s reported symptoms, an upper gastrointestinal endoscopy was performed to evaluate the possible presence of esophageal stricture and a foreign body (**Figure 3**, upper panels). The endoscopy revealed circumferential esophageal candidiasis at the entrance and upper part of the esophagus, with the middle portion being completely obstructed by a piece of chicken meat and other food debris. The chicken meat was successfully crushed with endoscopic forceps and removed from the esophagus by pushing it into the stomach. In addition, the gastroenterologist suspected eosinophilic esophagitis and performed a biopsy at the time of follow-up endoscopy. However, there were no findings indicating the presence of eosinophilic infiltration.

Following one day of fasting, the patient was able to gradually resume diet, and after a week, a follow-up endoscopy was performed without any specific incident (**Figure 3**, lower panels), leading to discharge. Since he was a very fast eater, we performed nutritional guidance for him to eat slowly and chew his food well. Finally, he was placed in a facility that provided meals because he was elderly and lived alone, and after then, no similar symptoms were observed.

## Discussion

In this report, we show a case of repeated esophageal obstruction in a patient with Type 3C DM complicated by advanced and severe diabetic neuropathy. Although diabetic neuropathy is a well-known cause of gastrointestinal motility disorders, including gastroparesis, diarrhea, and constipation in intestinal enteropathy (4), it is rare that diabetic neuropathy brings about esophageal obstruction. Despite the limited attention given to esophageal motor disorders in the complications of DM, their reported prevalence is as high as 63%, surpassing that of gastroparesis (5).

Esophageal symptoms of diabetic neuropathy, including abnormal peristalsis, spontaneous contractions, and impaired lower esophageal sphincter tone, can lead to symptoms such as heartburn and dysphagia (6, 7). These symptoms have been reported in 5.4% of DM patients and are commonly attributed to an esophageal motor disorder (8). The pathophysiology of esophageal symptoms in DM patients is complex and multi-factorial, and is thought to involve a combination of hyperglycemia, autonomic neuropathy, biomechanical and sensory alterations of the esophagus, presbyesophagus, and psychiatric comorbidities (9, 10). The identification of esophageal dysfunction in DM patients is clinically significant, as it has been linked to delayed transit of food and oral medications (11, 12).

Several studies have indicated that patients with type 1 DM have a lower incidence of gastrointestinal symptoms in comparison to the general population (13). This may be attributed to esophageal hypo-sensitivity due to long-standing diabetic neuropathy. Additionally, it has been established that esophageal transit is delayed in approximately half of patients with long-standing DM (14). Type 3C DM, which is similar to type 1 DM, may result in poor sensitivity to neurological symptoms as a result of continued poor glycemic control and progressive complications.

There is a limitation in this case reports. Esophageal candidiasis may cause esophageal stricture. Esophageal candidiasis has been recognized more frequently in immunocompromised hosts, as the result of the examination of esophagogastric endoscopy (15). While esophageal research in diabetics focuses mainly on motility dysfunction, DM patients are at a higher risk for a number of other esophageal disorders (16). It has been reported that in several cases underlying diabetes was the only predisposing factor for esophageal candidiasis (17–19). However, since follow-up endoscopy showed the improvement of esophageal candidiasis without antifungal drugs, we think the main pathology of this patient was gastrointestinal motility disorders induced by diabetic neuropathy. We discussed the possibility of esophageal achalasia or gastroparesis in this case with the gastroenterologist; however, based on imaging examination and clinical time course, we finally diagnosed this subject as esophageal obstruction by diabetic neuropathy. In addition, according to the patient, he drank alcohol only occasionally after the onset of acute pancreatitis, but esophageal stricture in this case may have been caused by alcoholic neuropathy.

Taken together, we should bear in mind that impaired esophageal transit is a complication of severe diabetic neuropathy in patients with DM. Furthermore, if severe diabetic neuropathy persists in the condition of continued poorly controlled DM for a long period of time, it can lead to esophageal obstruction with limited subjective symptoms.

## Data availability statement

The original contributions presented in the study are included in the article/supplementary material. Further inquiries can be directed to the corresponding author.

## Ethics statement

Written informed consent was obtained from the individual(s) for the publication of any potentially identifiable images or data included in this article.

## Author contributions

TA researched data and wrote the manuscript. KaK, HT and TK researched data and contributed to the discussion. KoK and HK reviewed the manuscript. All authors contributed to the article and approved the submitted version.
